# Metabolically active brown adipose tissue in PPGL: an observational cohort study

**DOI:** 10.1530/ERC-24-0200

**Published:** 2025-03-07

**Authors:** Eduard Oštarijaš, Michael C Onyema, Zoulikha Zair, David R Taylor, Fannie Lajeunesse-Trempe, Saira Reynolds, Nicola Mulholland, Ben Corcoran, Mohamed Halim, Eftychia E Drakou, Ashley B Grossman, Royce P Vincent, Simon J B Aylwin, Georgios K Dimitriadis, Silvija Canecki-Varžić

**Affiliations:** ^1^ Doctoral School of Clinical Medical Sciences, Medical School, University of Pécs, Pécs, Hungary; ^2^ Department of Endocrinology ASO/EASO COM, King’s College Hospital NHS Foundation Trust, London, UK; ^3^ Department of Clinical Biochemistry (Synnovis), King’s College Hospital NHS Foundation Trust, London, UK; ^4^ Quebec Heart and Lung Institute, Quebec City, Canada; ^5^ Department of Nuclear Medicine, King’s College Hospital NHS Foundation Trust, London, UK; ^6^ Department of Clinical Oncology, Guy’s Cancer Centre – Guy’s and St Thomas' NHS Foundation Trust, London, UK; ^7^ Green Templeton College, University of Oxford, Oxford, UK; ^8^ Centre for Endocrinology, William Harvey Institute, Barts and The London School of Medicine and Dentistry, London, UK; ^9^ Neuroendocrine Tumour Unit, Royal Free Hospital, London, UK; ^10^ Faculty of Life Sciences and Medicine, School of Cardiovascular and Metabolic Medicine & Sciences, King’s College London, London, UK; ^11^ Department of Pathophysiology, Josip Juraj Strossmayer University of Osijek School of Medicine, Osijek, Croatia

**Keywords:** brown adipose tissue, BAT, phaeochromocytoma, paraganglioma, PPGL, FDG-PET, catecholamines

## Abstract

Brown adipose tissue (BAT) activity, identifiable through fluorodeoxyglucose positron emission tomography (FDG-PET), has gained interest due to its potential link with metabolic disorders and tumour pathophysiology. This study aims to explore the activation of BAT in patients with phaeochromocytoma/paraganglioma (PPGL) and its clinical relevance. This retrospective observational study, conducted in a large academic centre in London, reviewed FDG-PET images of 62 confirmed PPGL patients, collected between 2013 and 2021. We assessed patient demographics, biochemistry, radiological features, mutational status and outcomes, focussing on activated BAT detection. Of the 62 patients, 13% demonstrated active brown adipose tissue (aBAT) on FDG-PET imaging. Histopathological confirmation of BAT from one patient was used to validate BAT activation observed during imaging. Multivariate analysis indicated that elevated plasma normetanephrine concentrations were directly proportional to aBAT presence, suggesting their strong association with BAT activation. Despite identifying aBAT, no significant differences were found in BMI, sex, age or mutational status between aBAT-positive and aBAT-negative groups. Kaplan–Meier survival plots assessing overall and progression-free survival did not reach statistical significance. This study underscores the complex interaction between catecholamine excess and BAT activation in patients with PPGLs. The findings suggest that aBAT activity might be an indicator of severe catecholamine excess (especially normetanephrine), potentially influencing patient outcomes. Our study adds to the limited pool of knowledge and offers novel insights into BAT activation in patients with PPGLs, highlighting its potential link with metabolic derangements and patient outcomes.

## Introduction

White adipose tissue (WAT) and brown adipose tissue (BAT) form the main adipose tissue subtypes in humans and several animal species. Recently BAT, owing to its unique metabolic function, has been of increased focus and interest in metabolic research (Santhanam *et al.* 2015). BAT is the major organ of non-shivering thermogenesis in the body, with this activity being dependent on the large number of mitochondria and increased expression of uncoupling protein-1 (UCP-1) activity present within this type of tissue (Fenzl & Kiefer 2014). There are numerous triggers for the metabolic activation of BAT, including cold temperature, low body mass index (BMI), adrenergic agonists and elevated concentration of thyroid hormones (Marlatt & Ravussin 2017).

Clinical research suggests that activation and thermogenesis in BAT is mediated by norepinephrine release from the sympathetic nervous system (Bartness *et al.* 2010). BAT has traditionally been considered to mainly express β_3_-adrenoreceptors; however, *in vitro* studies have indicated that activated β_2_-adrenoreceptors may be the main driving force behind thermogenesis (Blondin *et al.* 2020). Active brown adipocytes take up glucose from the circulation, which they use to synthesise free fatty acids (Jung *et al.* 2019). Due to this aspect of BAT metabolism and the increasing use of fluorodeoxyglucose positron emission tomography (^18^FDG-PET) imaging, there has been an increased detection rate of activated brown adipose tissue (aBAT); this may affect diagnoses by leading to false-positive reporting of tumours (Nedergaard *et al.* 2007).

BAT is most abundant in foetuses and infants, with significant regression of levels into adulthood. In newborn infants, BAT is located in large interscapular and perirenal depots, while in adults, BAT is mainly located in the neck, mediastinum, axilla, retroperitoneum and abdominal wall (Iyer *et al.* 2009).

Phaeochromocytomas/paragangliomas (PPGLs) are catecholamine-producing endocrine tumours that emerge from the adrenal medulla or extra-adrenal ganglia. High FDG accumulation in aBAT has been frequently noted in patients with PPGL, with subsequent resolution of these findings after PPGL resection (Terada *et al.* 2019). This finding is likely related to the increased glucose transport related to norepinephrine excess (Iyer *et al.* 2009).

Studies reviewing PPGLs have shown an aBAT detection rate of 7.8–42.8% on FDG-PET imaging, correlating with elevated catecholamine levels but without clear correlation to germline mutations (Hadi *et al.* 2007, Wang *et al.* 2011, Puar *et al.* 2016, Abdul Sater *et al.* 2020). In one study, this imaging finding was associated with a statistically significant reduction in overall survival (OS) (Abdul Sater *et al.* 2020). Standardisation for standardised uptake value (SUV) cut-offs for aBAT on FDG-PET are lacking, but these are often reported between 1.0 and 2.0 (Sampath *et al.* 2016); in previous studies of PPGL, a cut-off value of >1.5 has been employed (Puar *et al.* 2016, Abdul Sater *et al.* 2020).

Research on the clinical implications of aBAT in patients with PPGL remains scarce. The main objectives of this study were to gain further insights into BAT activation rates in patients with PPGLs and how this may relate to patient demographics, biochemistry, radiological features, mutational status and outcomes. The main hypotheses were that aBAT rates would be significantly linked to the severity of catecholamine excess and could be considered a poor prognostic feature.

## Methods

### Study design

This is a retrospective observational study across a single tertiary academic institution (King’s College Hospital NHS Foundation Trust, UK) evaluating the presence of aBAT in patients who had undergone FDG-PET imaging for suspected PPGL. The selection criteria for FDG-PET included patients with presumed disseminated or extra-adrenal disease or those with intermediate lesions based on strong clinical or radiological suspicion (e.g., findings on CT imaging). These cases were reviewed in adrenal multidisciplinary team meetings, where the indication for FDG-PET was determined. Guideline recommendations for imaging in PPGL were followed (Fassnacht *et al.* 2016).

### Patient characteristics and clinical assessment

The following data were collected for each PPGL patient: demographics, baseline biochemistry (plasma metanephrine (pmol/L), normetanephrine (pmol/L) and 3-methoxytyramine (pmol/L)), baseline imaging findings (anatomical location of aBAT, total number of lesions, size of largest lesion and presence of distant metastases), histology, mutational status, treatment modality, response evaluation criteria in solid tumours (RECIST) v1.1 criteria response to treatment (Eisenhauer *et al.* 2009), progression-free survival (PFS) and OS.

The PPGL patients included had their baseline FDG-PET scans reviewed by two nuclear medicine consultant physicians experienced in FDG-PET CT reporting. Cases were independently scored for the presence of aBAT in predetermined locations (supraclavicular, paravertebral or perirenal). Any discrepant cases were mediated by a third such experienced nuclear medicine consultant physician. Images were transferred from the picture archiving and communication system to the HERMES workstation to allow dedicated analysis/SUV quantification. In cases with multiple scans, we included the first-ever FDG-PET scan that detected aBAT in patients with PPGL for our analysis.

### Statistical analysis

Continuous data were assessed for normality using the Shapiro–Wilk test. Normal data were expressed as the mean value with its respective standard deviation (*A*_r_ ± SD). Non-normal data were presented as the median with interquartile range (MED (IQR)). We reported categorical data using ratios and percentages. Missing data within the dataset were imputed using the *missRanger* package (version 2.4.0), employing a random forest algorithm for iterative imputation. The function was iterated 1,000 times, with each random forest consisting of 100 trees.

To investigate the potential association of covariates with the incidence of aBAT, we conducted a multivariate analysis using a logistic regression. The model was fitted with Firth’s bias reduction method to address issues of small sample sizes and rare events. The analysis incorporated pre-defined clinical covariates, while the results were presented as adjusted odds ratios for each covariate, with their respective 95% confidence intervals (95%–CI) and *P*-values (*α* = 0.05) alongside non-adjusted (crude) ORs. The *logistf* package (version 1.26.0) was used for the multivariate logistic regression analysis.

To assess statistical significance in difference of observed numerical variables, we implemented an R function for non-parametric bootstrap *P*-value calculation (Dwivedi *et al.* 2017) due to relatively small sample size. The function performed iterative resampling (10,000 iterations) to compute t-statistics for two-group comparisons, determining the *P*-value based on the proportion of resampled statistics. Statistical significance of independence of categorical data was tested using Fisher’s exact tests for count data.

We produced Kaplan–Meier plots to compare the OS and PFS times between the investigated and control group. Due to the sample size, the significance of survival differences was assessed using a univariate Cox model.

All analyses were performed in the R version 4.4.0.

### Ethics

Ethical approval from a Regional Ethical Committee (REC) in the United Kingdom (UK) was not required as the data generated for the purposes of this project were fully anonymised, collected in line with the standard of care protocols for treating patients with PPGL at the King’s College Hospital, and are processed and presented retrospectively. The study was discussed within research delivery unit 6 (RDU6) meeting – Renal/Endo Research Group Board (RRGB) at the King’s College Hospital NHS Foundation Trust (Governance Arrangements for Research Ethics Committees (GafREC): Endo203). The study is registered with clinicaltrial.gov, with registration number NCT06440122.

## Results

An initial list of 93 patients with suspected PPGL having undergone FDG-PET imaging was obtained from the nuclear medicine department covering the time period from 2013 to 2021. After exclusion of duplicate patients and reviewing electronic notes for the inclusion of only those with a PPGL diagnosis confirmed after discussion in multidisciplinary team meetings, this list was reduced to 62 patients (22 male, 40 female).

**Figure 1:**
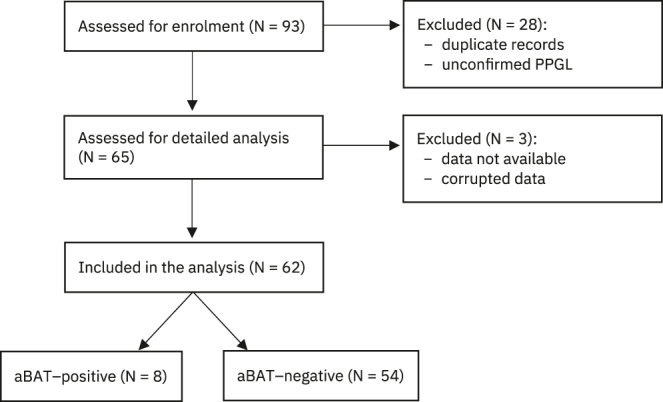
Enrolment and analysis flowchart.

Three cases were excluded at the stage of dedicated analysis/SUV quantification (two cases had corrupted data and one case was not available) and were excluded from subsequent analyses. Sixty-two cases were subsequently scored. Fifty-four cases (87%) were deemed not to have aBAT (53 initial agreement and one mediation) and eight cases (13%) were deemed to have aBAT (seven initial agreement and one mediation) (Fig. 1). The cases without aBAT would form the basis of unmatched controls for further statistical analysis.

**Figure 2:**
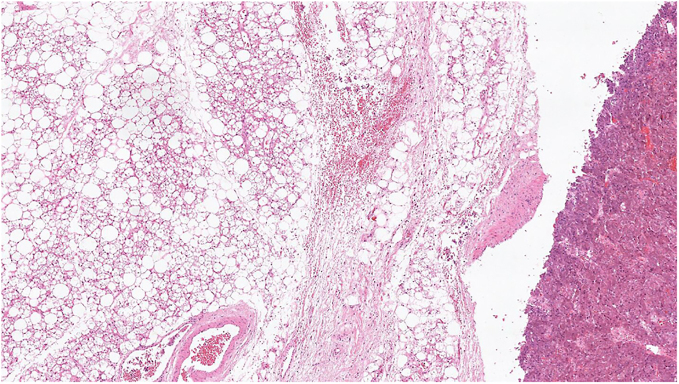
Low magnification image shows phaeochromocytoma to the far right of the image in an aBAT patient from our cohort in a retrospective post-surgical specimen. BAT adjacent to the phaeochromocytoma is seen occupying the left two-thirds of the field.

**Figure 3:**
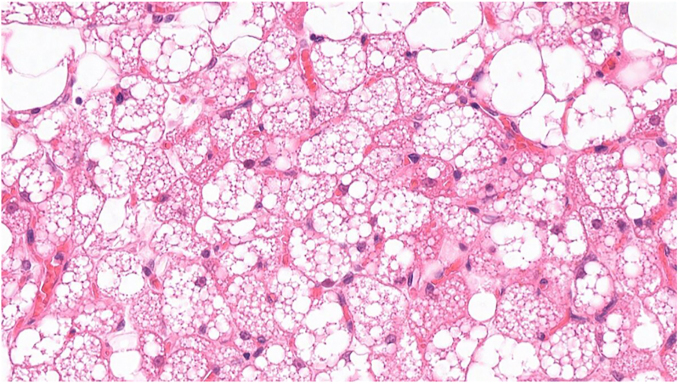
At higher magnification (from Fig. 2), the adipocytes in brown fat show multivacuolated cytoplasm resulting from numerous small lipid droplets. The cytoplasm is acidophilic and granular due to large number of mitochondria. Small number of white fat adipocytes are present in this field for comparison. These have a single intracytoplasmic large fat droplet only.

**Figure 4:**
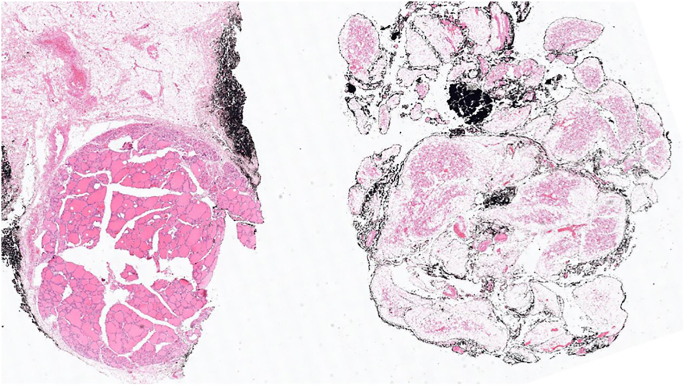
Low magnification shows thyroid tissue on the left-hand side of the field and brown fat in the adjacent right-hand side of the field.

One of our patients in the aBAT-positive cohort had a confirmed diagnosis of MEN 2A proceeding to a right adrenalectomy for phaeochromocytoma and total thyroidectomy for medullary thyroid carcinoma (MTC), both in 2014 with satisfactory surgical clearance. Areas of aBAT uptake on FDG-PET were retrospectively confirmed (in supraclavicular, paravertebral, perirenal and ‘other’ locations with highest maximum standardised uptake value (SUV_max_) of 10.6) and correlated with BAT on histology specimens (Figs 2, 3, 4).

The results of demographic and clinical data analysis and laboratory, imaging, pathology, treatment and follow-up information are presented in the patient characteristics table (Table 1) with respect to the presence or absence of aBAT. Except for the plasma metanephrine concentration, which was significantly negatively associated with aBAT, the differences in categorical and continuous data between the groups did not reach statistical significance in the univariate analysis. While statistically insignificant, the aBAT-positive group showed trends (defined as *P* < 0.2) in association with lower age, female sex, lower BMI, absence of prior cardiovascular disease, lower number of antihypertensive medication and lower OS. In our study, although no patients with detectable aBAT were taking adrenoreceptor blockade, we found no statistical association between aBAT and either alpha- or beta-blocking medication (*P* = 0.581 and *P* = 1.000, respectively). Detailed per-patient data for the aBAT-positive group are available separately in Table 2.

**Table 1:** Patient characteristics table.

	aBAT-positive (*n* = 8)	aBAT-negative (*n* = 54)	Pre-imputation data availability	*P*-value
Demographic information				
Age (years)	46.0 (29.8, 51.0)	54.5 (39.8, 60.8)	61/62 (98.4%)	0.126
Sex			62/62 (100%)	0.240
Female	7 (87.5%)	33 (61.1%)		
Male	1 (12.5%)	21 (38.9%)		
Clinical data				
BMI (kg/m^2^)	25.8 ± 4.82	26.9 ± 5.23	51/62 (82.3%)	0.548
Prior cardiovascular disease	0 (0%)	13 (24.1%)	59/62 (95.2%)	0.186
Hypertension	2 (25%)	29 (53.7%)	59/62 (95.2%)	0.255
Number of antihypertensives	0.389 ± 0.737	0.903 ± 1.090	57/62 (91.9%)	0.083
Alpha-blockade	0 (0%)	8 (14.81%)	59/62 (95.2%)	0.581
Beta-blockade	0 (0%)	5 (9.26%)	59/62 (95.2%)	1.000
Positive family history	1 (12.5%)	6 (11.1%)	56/62 (90.3%)	1.000
Laboratory parameters				
Plasma metanephrine (pmol/L)	279 (202, 913)	505 (169, 1,920)	57/62 (91.9%)	0.034
Plasma normetanephrine (pmol/L)	7,740 (674, 14,700)	2,320 (1,010, 10,100)	58/62 (93.6%)	0.434
Ratio of plasma normetanephrine/metanephrine	14.3 (3.66, 25.90)	6.45 (3.03, 17.70)		0.472
Plasma 3-methoxytyramine (pmol/L)	131 (120, 231)	120 (120, 232)	56/62 (90.3%)	0.876
Imaging and pathology				
Number of aBAT locations	3.90 ± 2.78	Not applicable	8/8 (100%)	
Highest aBAT SUV_max_	5.69 (3.87, 12.70)	Not applicable	8/8 (100%)	
Tumour type			58/62 (93.6%)	1.000
Phaeochromocytoma	5 (62.5%)	34 (63.0%)		
Paraganglioma	3 (37.5%)	20 (37.0%)		
Size of largest tumour (mm)	57.5 (48.7, 70.3)	49.5 (30.0, 64.3)	54/62 (87.1%)	0.876
Cluster			62/62 (100%)	1.000
No mutation	4 (50.0%)	17 (31.5%)		
Cluster 1 (SDHx, VHL)	1 (12.5%)	9 (16.7%)		
Cluster 2 (MEN, RET)	0 (0%)	2 (3.7%)		
Unknown	3 (37.5%)	26 (48.1%)		
AJCC staging			57/62 (91.9%)	0.374
1	1 (12.5%)	16 (29.6%)		
2	1 (12.5%)	14 (25.9%)		
3	3 (37.5%)	8 (14.8%)		
4	3 (37.5%)	16 (29.6%)		
Metastatic disease	3 (37.5%)	16 (29.6%)	57/62 (91.9%)	0.692
Treatment				
Chemotherapy	1 (12.5%)	4 (7.41%)	59/62 (95.2%)	0.511
Surgery	6 (75%)	46 (85.2%)	60/62 (96.8%)	0.604
Radiotherapy	0 (0%)	3 (5.56%)	60/62 (96.8%)	1.000
MIBG therapy	1 (12.5%)	8 (14.8%)	59/62 (95.2%)	1.000
Follow-up				
RECIST v1.1 criteria			62/62 (100%)	0.292
CR	1 (12.5%)	11 (20.4%)		
PR	0 (0%)	6 (11.1%)		
SD	0 (0%)	2 (3.7%)		
PD	3 (37.5%)	5 (9.3%)		
No data	4 (50.0%)	30 (55.6%)		
Mortality	2 (25.0%)	12 (22.2%)	62/62 (100%)	1.000
PFS (months)	41.0 (22.5, 60.0)	36.0 (24.3, 55.3)	62/62 (100%)	0.913
OS (months)	41.0 (23.3, 60.8)	47.5 (32.8, 95.3)	62/62 (100%)	0.136

Non-normal data presented as median with interquartile range (MED (IQR)). BMI, body mass index; PFS, progression-free survival; OS, overall survival; aBAT, active brown adipose tissue; SUV_max_, maximum standardised uptake value; RECIST, response evaluation criteria in solid tumours; CR, complete response; PR, partial response; SD, stable disease; PD, progressive disease.

**Table 2:** Individual characteristics of patients with aBAT.

Patient	Age	Sex	BMI (kg/m^2^)	Pl. metanephrine (pmol/L)	Pl. normetanephrine (pmol/L)	Pl. 3-methoxytyramine (pmol/L)	Hypertension	Highest SUV_max_	Tumour type	Size of largest tumour (mm)	Mutational status	Metastatic disease	Family history	Mortality	RECIST v1.1
1	45	F	26.2	56	337	142	No	3.88	PCC	49	N/A	No	−	No	ND
2	29	M	24.6	1,766	>40,000	235	No	10.60	PCC	71	W/T	No	−	No	ND
3	61	F	28.6	134	<109	<120	No	3.39	PGL	70	N/A	Yes	−	Yes	PD
4	49	F	N/A*	224	14,476	677	Yes	4.09	PCC	65	W/T	Yes	−	Yes	PD
5	28	F	22.1	1,966	7,945	<120	No	23.40	PCC	46	W/T	No	−	No	CR
6	47	F	35.5	314	786	<120	Yes	7.29	PCC	49	N/A	No	−	No	ND
7	57	F	20.8	243	7,544	<120	No	3.83	PGL	50	W/T	No	−	No	ND
8	30	F	21.4	629	15,227	229	No	18.81	PGL	86	SDHB	Yes	+	No	PD

BMI, body mass index; M, male; F, female; SUV_max_, maximum standardised uptake value; PCC, phaeochromocytoma; PGL, paraganglioma; N/A, not available; W/T, wild-type; SDHB, succinate dehydrogenase complex subunit B; RECIST, response evaluation criteria in solid tumours; ND, no data; PD, progressive disease; CR, complete response; aBAT, active brown adipose tissue.

* Patient no. 4’s BMI was recorded as 12.6 kg/m^2^. Due to concerns about the reliability of this value, we initially included their anthropometric data, but encountered difficulties in validating it, despite multiple attempts. While such a low BMI is clinically plausible, we were unable to confirm its accuracy through available clinical notes. To address this, we performed an imputation analysis to evaluate its plausibility in the context of other parameters for this patient. Ultimately, we treated this value as missing data and excluded it from the analysis. This adjustment did not affect the statistical significance in BMI difference between the groups.

**Figure 5:**
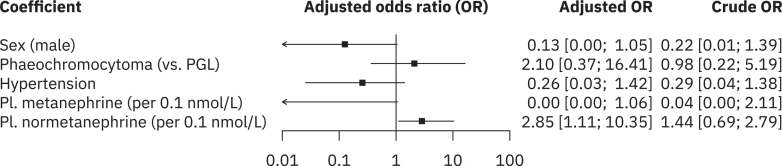
Forest plot showing multivariate adjusted and unadjusted odds ratios of aBAT presence associated with multiple covariates.

Penalised multivariate analysis with multiple clinical covariates indicated that male sex (adjusted OR 0.1; CI 0.00, 1.05), tumour type (adjusted OR 2.10; CI 0.37, 16.41), hypertension (adjusted OR 0.26; CI 0.03, 1.42) and increased plasma metanephrine levels (adjusted OR 0.00; CI 0.00, 1.06) were not significantly associated with the presence of aBAT. In contrast, increased plasma normetanephrine levels (adjusted OR 2.85; CI 1.11, 10.35) showed a statistically significant trend towards aBAT presence, as shown in Fig. 5.

**Figure 6:**
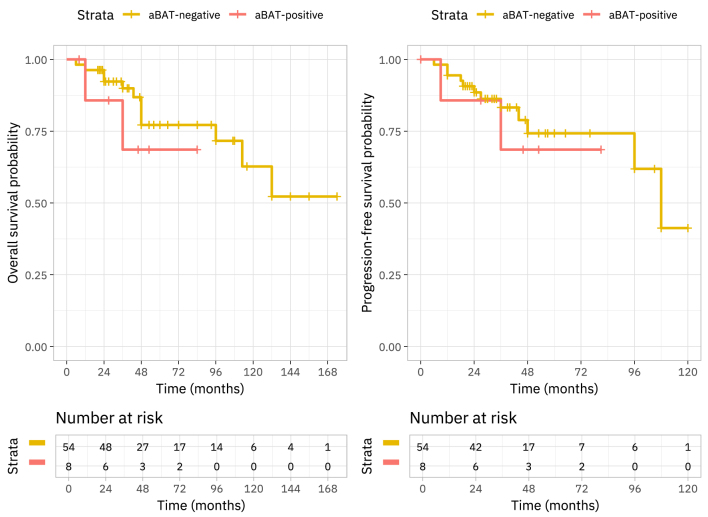
Kaplan–Meier survival plots: (A) OS, (B) PFS.

The univariate Cox proportional hazards model indicated hazard ratios of 1.77 (CI 0.38, 8.18) and 1.38 (CI 0.30, 6.34) for overall and PFS probabilities, respectively. The survival data for both overall and PFS are visually represented using Kaplan–Meier survival curves in Fig. 6.

## Discussion

The main objectives of our study were to investigate BAT activation rates detected by FDG-PET and its relationship with patients’ characteristics, biochemistry, radiological features, mutational status and outcomes in patients with PPGLs.

Previous studies reviewing PPGLs have reported on probable BAT activity in patients with PPGL. Specifically, an aBAT prevalence of 7.8–42.8% was observed on FDG-PET imaging using less stringent criteria of SUV_max_ cut-offs for aBAT between 1.0 and 2.0; in other cases, activity of BAT was quantified by mean standardised uptake values (SUV_mean_) (Hadi *et al.* 2007, Wang *et al.* 2011). We found that 13% of PPGL patients had aBAT positivity on FDG-PET imaging (*n* = 8/62) using a stricter criterion of SUV_max_ cut-off value of 1.5 in line with more recent findings (Puar *et al.* 2016, Abdul Sater *et al.* 2020).

However, FDG-PET analysis alone does not confirm BAT, but only suggests its presence due to its correlation with anatomical area of specific metabolic activity. One of our patients in the aBAT-positive cohort with a confirmed diagnosis of MEN 2A underwent right adrenalectomy for phaeochromocytoma and total thyroidectomy for MTC. BAT adjacent to the phaeochromocytoma and MTC was histopathologically confirmed in line with the FDG-PET findings, providing a direct validation of BAT activation.

To the best of our knowledge, this is the first clinical study assessing BAT activation on FDG-PET and its clinical implications in PPGL patients with histopathological confirmation of BAT. Albeit on a single patient, the pathological confirmation supports the correlation between imaging findings and true aBAT in our study. Furthermore, unlike other clinical cohort papers assessing aBAT in PPGL patients, our study incorporates a robust multivariate statistical approach, additionally contributing to the generalisability of implications.

From a methodological and statistical standpoint, the main limitations of this study arise from its retrospective nature and the relatively small number of cases. While we histopathologically confirmed aBAT in one patient, consistent histological correlation across multiple cases was not feasible due to the ethical and practical constraints of obtaining multiple biopsies from patients in clinical and surgical practice. In addition, the retrospective observational design of our study limited the ability to systematically perform such analyses. As with all cohort studies, it is also possible that transient or persistent confounding factors that could have affected the results were overlooked. Despite having access to electronic records, the information was not always comprehensive for some out-of-region cases, leading to known or unknown missing data points. However, to ensure robust imputation, we employed a random forest algorithm for iterative imputing using 1,000× iterations, with each random forest consisting of 100 trees. Nevertheless, because of relatively low sample size given the rarity of this group of neoplasms and the less common use of FDG-PET imaging to characterise them, the implied trends in our study should be interpreted with caution.

Regarding the clinical characteristics of patients and BAT activation, our study did not reveal significant differences between the aBAT-positive and aBAT-negative groups in terms of BMI, sex or age; although age is often considered the strongest determinant of BAT activation (Pfannenberg *et al.* 2010, Puar *et al.* 2016), the limited age range for these patients may not have identified this.

The inclusion of our study’s results in a recently performed cumulative meta-analysis has further validated our findings (Onyema *et al.* 2024). The meta-analysis revealed a statistically significant positive association between demethylated catecholamine levels and the presence of aBAT, while the inclusion of our study reduced overall heterogeneity in the pooled data, particularly in terms of catecholamine level variability. Sensitivity analyses, conducted with and without our data, demonstrated that our findings reduced heterogeneity and reinforced the observed trends. This confirms the reliability of our results across analytical scenarios, despite the initial non-significance of normetanephrine levels in our univariate analysis. By contributing to a larger evidence pool, our study enhanced the statistical robustness of the meta-analysis, offering a more robust perspective on aBAT prevalence and its biochemical correlates. Animal studies have shown that norepinephrine stimulation of BAT *via* β_3_-receptors leads to an increased number of BAT cells, lipolysis, glucose transportation, uncoupling protein-1 (UCP1) expression, and ultimately, thermogenesis (Cannon & Nedergaard 2004, Puar *et al.* 2016). Significant BAT activation in patients with PPGLs is likely to be due to the systemic effects of catecholamine excess, as reported in previous studies (Puar *et al.* 2016). In our study, average normetanephrine concentration was higher in the aBAT-positive group, which was statistically significant in the multivariate analysis, although this difference did not reach the limit of significance in the univariate analysis of the differences in medians. Plasma metanephrine levels, however, showed statistical significance in the univariate analysis, inversely proportional to the presence of aBAT, indicating a potentially strong preference for increased normetanephrine/metanephrine ratio’s association with BAT activation. Although norepinephrine is typically associated with both phaeochromocytomas and paragangliomas (unlike epinephrine, which is produced only in phaeochromocytomas) (Al-Harthy *et al.* 2009), our study had comparable numbers of patients with phaeochromocytomas and paragangliomas associated with activated BAT. Since aBAT is a highly vascularised and metabolically active tissue, it could facilitate the clearance of circulating catecholamines. It may uptake and metabolise catecholamines as part of its thermogenic activity, potentially reducing the demethylated catecholamine pool available for conversion into metanephrines. In addition, the aBAT-positive group might represent a subgroup with a distinct hormonal or tumour phenotype leading to an altered catecholamine production or metabolism.

In addition, 86% of our patients were deemed to not have aBAT. This raises the question of whether there are other mechanisms of BAT activation beyond sympathetic stimulation and what are the other ‘browning’ factors in PPGL. BAT activation has previously been linked to malignancies (Santhanam *et al.* 2015), and clinical studies have reported characteristic alterations in BAT in patients with cancer (Dong *et al.* 2023). Evidence regarding the role of catecholamine excess in BAT activation in these patients is controversial. The mechanisms underlying dynamic interactions between cancer cells and stromal adipocytes remain unclear (Paré *et al.* 2020). It is generally believed that BAT predominantly expresses β_3_-adrenoreceptors and exhibits enhanced norepinephrine responsiveness compared to WAT (Cero *et al.* 2021). However, *in vitro* experiments indicate that thermogenesis in BAT might primarily be mediated through the stimulation of β_2_-adrenoreceptors (Blondin *et al.* 2020). Although no patients with detectable aBAT in our study were taking adrenoreceptor blockade, the lack of statistical association between aBAT and either alpha- or beta-blocking medication (*P* = 0.581, *P* = 1.000, respectively) suggests that these medications were unlikely to confound our findings. However, the possibility of an undetected influence cannot be completely excluded.

BAT activation, including browning of WAT to form beige adipocytes, can be induced by multiple factors including thyroxine, bile acids and factors overexpressed in certain malignant tumours, including interleukin 6 (IL-6), parathyroid hormone-related peptide (PTH-rP), fibroblast growth factor 21 and natriuretic peptides (Villarroya & Vidal-Puig 2013, Abdul Sater *et al.* 2020). PPGLs, while principally being known for secretion of catecholamines, can also secrete various growth factors responsible for autocrine or paracrine functions (Kontogeorgos *et al.* 2002, Abdul Sater *et al.* 2020). Adrenomedullin (ADM), a vasodilator peptide hormone induced by hypoxia, was initially isolated in 1993 from a phaeochromocytoma (Kitamura *et al.* 1993). In a three-dimensional mammosphere model, breast cancer cells secrete adrenomedullin to promote lipolysis and browning of adjacent mature adipocytes, which highly express the adrenomedullin receptors (Paré *et al.* 2020, Dong *et al.* 2023). Further studies are needed to investigate the association between PPGLs and browning factors other than catecholamines.

Anatomically, many types of solid tumours (e.g., breast cancer, prostate cancer and renal cancer) grow in either direct contact or close proximity to adipose tissue, which provides a suitable model for studies investigating the direct interactions between cancer cells and adipocytes (Dong *et al.* 2023). Tumour cells interact with the cells that compose their environment to promote tumour growth and invasion. Among them, adipocytes provide lipids that are used as a source of energy and adipokines that contribute to tumour expansion (Paré *et al.* 2020, Dong *et al.* 2023). Although beneficial in some other circumstances, such as obesity and diabetes prevention, BAT was found to contribute to the development of complications, such as cancer-induced cachexia (Nishio & Saeki 2020, Jalloul *et al.* 2023). It was hypothesised as early as 1981 that BAT activation induces a hypermetabolic state and contributes to weight loss in cancer patients (Ma *et al.* 2015, Jalloul *et al.* 2023). Recent studies have emphasised the potential implication of BAT activity in the weight status of oncological patients and demonstrated that localisation, along with the amount of activated BAT, could influence its effects on BMI (Jalloul *et al.* 2023). Moreover, a recently published case report described a patient with phaeochromocytoma and BAT activation, evidenced by significant FDG uptake that resolved postoperatively alongside normalisation of plasma catecholamine levels (Md Nor *et al.* 2024).

In our study, we found that increased plasma normetanephrine concentration were associated with active BAT. We did not find that BAT activation was significantly associated with any assessed imaging findings, other pathological findings or treatment modalities. BAT activation was also not associated with specific germline mutations, which is consistent with previous findings (Puar *et al.* 2016). In our study, we observed a greater proportion in advanced AJCC staging in aBAT-positive patients. Specifically, 75% of aBAT-positive patients were classified as stage 3 or 4, compared to only 44.4% of aBAT-negative patients, although this difference did not reach statistical significance. This disparity in tumour stage is possible to contribute to the shorter survival rates observed in aBAT positive patients. Hence, we aimed to account for tumour stage in the multivariate analysis or compare the eight aBAT-positive subjects to a matched control cohort, but the small sample size limits the statistical power and interpretability of such comparisons in the context of survival analysis.

There is growing interest in prognostic biomarkers that can predict metastatic disease and survival in patients with PPGL. In our study, Kaplan–Meier survival plots indicated trends towards reduced OS and PFS in the aBAT-positive group; however, due to the stricter SUV_max_ cut-off used in our study and small sample size, this information was statistically underpowered. Previous studies have shown that elevated levels of norepinephrine and the detection of BAT activity by FDG-PET in patients with PPGL were associated with decreased OS, with norepinephrine as an independent risk factor for mortality, likely unrelated to cardiovascular complications (Abdul Sater *et al.* 2020). Mechanistically, there is evidence suggesting that aBAT may confer poorer outcomes due to its contribution to a hypermetabolic state, which has been linked to cancer cachexia, malnutrition and metabolic derangements (Shellock *et al.* 1986, Beijer *et al.* 2012). It is well-established that severe catecholamine excess can exacerbate metabolic instability and increase cardiovascular disease risk – e.g., elevated catecholamine levels can lead to a catecholamine-induced cardiomyopathy in patients with phaeochromocytoma (Wilkenfeld *et al.* 1992, Zhang *et al.* 2017, Szatko *et al.* 2023). However, preclinical evidence indicates that aBAT activity may contribute to weight loss and cancer cachexia, a condition characterised by profound malnutrition and systemic metabolic disruption (Brooks *et al.* 1981). These factors could compromise patient resilience, potentially leading to lower performance scores, reduced ability to tolerate treatments and a diminished likelihood of surgical interventions leading to poorer survivability.

The role of catecholamines in tumour progression remains intriguing. Earlier studies have suggested that catecholamines directly affect tumour cell behaviour and gene expression by growth-promoting effects and possible chemotactic activity of norepinephrine-producing organs to recruit tumour cells (adrenal gland and brain are the common sites of metastasis for several sites of malignancies). In addition, catecholamines have been shown to have anti-apoptotic effects on cancer cells and may render tumour cells resistant to chemotherapeutic drugs (Yang 2010). The variable effects of catecholamines can potentially be explained by the variable expression of nine adrenergic receptor isoforms and other factors, including catecholamine effects on cancer cells versus immune or endothelial cells (Wackerhage *et al.* 2022).

In the context of PPGL, severe catecholamine excess and its systemic effects may not only activate BAT, but also further exacerbate the negative impact of aBAT activity. The mechanistic pathways involving hypermetabolism, cancer cachexia, malnutrition and cardiovascular risks support the plausibility of aBAT being an independent determinant of survival. However, further research (both preclinical and clinical) based on prospective studies is required to delineate these pathways more clearly in the context of aBAT in patients with PPGL.

## Conclusions

Based on complex association between catecholamine levels and activation of BAT in patients with PPGL, our study investigated the potential role of aBAT as a marker of catecholamine excess and potential subsequent clinical outcomes in these patients. While the presence of aBAT significantly correlated only with elevated plasma normetanephrine concentration, the observed trends towards reduced survival in aBAT-positive patients suggest a potential link between BAT activation and more aggressive disease state. Although small, our study and the used robust statistical approach enrich the limited pool of existing data, allowing for future secondary analyses and thereby adding significant value to the cumulative knowledge in this domain. Future research should focus on generating data from larger prospective cohort and clarifying the underlying mechanisms of BAT activation beyond sympathetic stimulation, exploring further metabolic and molecular ‘beiging’ factors in PPGL, and investigating the prognostic implications of BAT activity.

## Declaration of interest

EO received an honorarium from Krka. GKD received research grants from NIHR, Novo Nordisk, UK Research Foundation and DDM, and payment or honoraria for lectures, presentations, speakers’ bureaus, manuscript writing or educational events from Novo Nordisk, Rhythm Pharmaceuticals, J&J/Ethicon and Medtronic. SCV received research grants, payment or honoraria for lectures, presentations or educational events from Novo Nordisk, Eli Lilly, Medtronic and Krka. GKD, EO and SCV report that there is no conflict of interest that could be perceived as prejudicing the impartiality of the work reported. Other authors did not report conflicts of interest.

## Funding

GKD received NIHR Southeast London Clinical Research Network (CRN) ‘Greenshoots’ Investigator Award, which allowed protected time to complete this work. This work did not receive any specific grant from any funding agency in the public, commercial or not-for-profit sector.
